# Unique divergence of the breast cancer 2 (*BRCA2*) gene in Neanderthals

**DOI:** 10.1186/s41065-018-0073-5

**Published:** 2018-11-03

**Authors:** Pawel Michalak, Lin Kang

**Affiliations:** 1 One Health Research Center, Virginia-Maryland College of Veterinary Medicine, 1410 Prices Fork Rd, Blacksburg, VA 24060 USA; 2 Edward Via College of Osteopathic Medicine, Blacksburg, VA 24060 USA; 3 Institute of Evolution, University of Haifa, Abba Khoushy Ave 199, 3498838 Haifa, Israel

**Keywords:** BRCA2, Tumor suppressor, Cancer, Neanderthal, Evolution

## Abstract

Unique divergence of the BRCA2, a tumor suppressor gene, in Neanderthals relative to other primates, including modern humans, is highlighted. This divergence with potentially pathogenic consequences raises a question about cancer susceptibility in the archaic species that was replaced by modern humans about 40,000 years ago.

## Main text

The discovery of tumor suppressor genes *BRCA1* and *BRCA2*, commonly known as breast cancer susceptibility genes, has been a major milestone in cancer research, diagnostics, and treatment. *BRCA1* and *BRCA2* are involved in the repair of DNA double-strand breaks and homologous recombination. High penetrance mutations in these genes result in a loss of tumor suppressor activity and increased risk of breast, ovarian, and, less frequently, other types of cancer [1]. Even though both genes have been known to evolve under rapid positive selection in extant primates [2], we highlight unusual sequence divergence of *BRCA2* in Neanderthals, the ancient relatives of modern humans.

The availability of two high coverage Neanderthal genomes [3, 4] enables their comparison with human and other primate genomes, which shows that Neanderthal *BRCA2* contains three unique non-synonymous nucleotide substitutions in the following positions of chromosome 13: (1) 32911273 (G2781A, M927I), (2) 32911463 (A2971G,N991D, Reference SNP rs1799944), and (3) 32913407 (G4915A, V1639I). All primate species that we used for comparison, i.e. Denisovan, another archaic species whose genome is available [5], modern human (hg19, Ensembl’s GRCh37.74), chimpanzee (CHIMP2.1.4.74), gorilla (gorGor3.1.74), orangutan (PPYG2.74), and gibbon (Nleu1.0.74), shared the same ancestral variant (human reference allele) in each of the three positions. The three Neanderthal loci were monomorphic, with sequence depth between 17 and 52x. Alleles in position (1) and (2) were shared by both Neanderthal individuals, while position (3) was monomorphic for the ancestral allele in the Vindija individual and polymorphic/heterozygous for the Neanderthal-specific allele in the Altai individual.

At least one of the Neanderthal variants (position (2)) is likely pathogenic, as the *BRCA2* SNP has been recorded by COSMIC database in association with two cancer types, haemangioblastoma and rhabdomyosarcoma [6]. However, functional consequences of these changes are unclear. Even though the two variants result in amino acid substitutions (M927I and V1639I), the protein structure is unaffected by these changes, as predicted using the AS2TS platform [7]. Based on the ‘1000 Genome’ database, the allele frequency is low (8%) and varies between populations from 3% (Europeans) to 13% (South Asians). Could thus the presence of this allele in modern humans be explained by interbreeding and introgression from Neanderthals? Only unlikely, as its frequency in Africans (5.5%) is not much lower than the global mean frequency. To our knowledge, the other two Neanderthal variants are absent from modern human populations altogether.

We have previously described more than a hundred other species-divergent substitutions in cancer-related genes that were prevalent germline genotypes in some primate species but in humans appeared only as somatic cancerous mutations [8]. These findings suggest that even closely related species may have evolved alternative counter-cancer adaptations, given that the same variant causes cancer in one species but not the other.

## Abbreviations

*BRCA2*: breast cancer 2 gene
COSMIC: Catalogue of Somatic Mutations in Cancer
